# Accuracy of Deep Learning in Detecting Cerebral Microbleeds: Systematic Review and Meta-Analysis

**DOI:** 10.2196/95041

**Published:** 2026-09-21

**Authors:** Yue Feng, Lei Zheng, Baiwen Zhang, Wei Zou

**Affiliations:** 1 Heilongjiang University of Chinese Medicine Harbin China; 2 Clinical Key Laboratory of Integrated Traditional Chinese and Western Medicine of Heilongjiang University of Chinese Medicine Harbin China; 3 The First Affiliated Hospital of Heilongjiang University of Chinese Medicine Harbin China

**Keywords:** cerebral microbleeds, deep learning, diagnosis, meta-analysis, systematic review

## Abstract

**Background:**

Traditionally, the number and location of cerebral microbleeds (CMBs) are manually calculated based on magnetic resonance imaging (MRI) characteristics such as shape, size, and signal features. Although accurate, manual detection requires expert interpretation and is costly. Therefore, it is necessary to explore an effective auxiliary detection method. In recent years, deep learning (DL) has been increasingly used in the detection of cerebral hemorrhage. Some studies have explored image-based DL models for diagnosing CMBs. Nevertheless, systematic evidence regarding their diagnostic accuracy is lacking.

**Objective:**

This review aimed to assess the accuracy of DL models in detecting CMBs and inform the development of intelligent detection tools.

**Methods:**

This study was reported in accordance with the PRISMA (Preferred Reporting Items for Systematic Reviews and Meta-Analyses) guidelines and was prospectively registered in PROSPERO (registration ID CRD42024628447). IEEE, Web of Science, Embase, the Cochrane Library, and PubMed were comprehensively searched up to November 1, 2024, and the database search was subsequently updated on July 5, 2026, to collect publicly published original studies on DL for detecting CMBs. The risk of bias of eligible studies was assessed using the Quality Assessment of Diagnostic Accuracy Studies-2 tool. Subgroup analyses were performed according to the level of analysis (lesion level and patient level) and the method of obtaining the diagnostic 4-fold table at the lesion level (direct extraction and reconstruction).

**Results:**

At the patient level, 5 studies were included, all of which developed models based on MRI. The meta-analysis results suggested that the sensitivity, specificity, positive likelihood ratio (PLR), negative likelihood ratio (NLR), and diagnostic odds ratio (DOR) were 0.89 (95% CI 0.76-0.96), 0.86 (95% CI 0.77-0.92), 6.3 (95% CI 3.5-11.6), 0.13 (95% CI 0.05-0.32), and 50 (95% CI 12-212), respectively. At the lesion level, the sensitivity, specificity, PLR, NLR, and DOR were 0.96 (95% CI 0.93-0.98), 0.98 (95% CI 0.94-0.99), 39.5 (95% CI 16.8-92.7), 0.04 (95% CI 0.02-0.07), and 1061 (95% CI 293-3848), respectively. In the subgroup of direct extraction of the diagnostic 4-fold tables at the lesion level, the sensitivity, specificity, PLR, NLR, and DOR were 0.98 (95% CI 0.95-0.99), 0.98 (95% CI 0.90-0.99), 40.2 (95% CI 9.3-79.9), 0.02 (95% CI 0.01-0.05), and 1738 (95% CI 174-17,385), respectively. In the subgroup of reconstruction of the diagnostic 4-fold tables at the lesion level, the sensitivity, specificity, PLR, NLR, and DOR were 0.95 (95% CI 0.89-0.97), 0.98 (95% CI 0.96-0.99), 41.2 (95% CI 21.2-79.9), 0.06 (95% CI 0.03-0.12), and 736 (95% CI 224-2418), respectively.

**Conclusions:**

DL models based on MRI appear to show favorable diagnostic performance in detecting CMBs. Given the small number of included studies, more multicenter studies are warranted to facilitate the development of more generalizable detection tools.

**Trial Registration:**

PROSPERO CRD42024628447; https://www.crd.york.ac.uk/PROSPERO/view/CRD42024628447

## Introduction

Cerebral microbleeds (CMBs) are characterized radiologically by small hypointense lesions in brain tissue on magnetic resonance imaging (MRI), particularly evident on susceptibility-weighted imaging (SWI) or T2*-weighted imaging (T2*WI). Histopathologically, CMBs refer to lesions caused by the perivascular accumulations of hemosiderin-laden macrophages within brain tissue [1,2]. The prevalence of CMBs varies significantly with detection methods and mean age. According to several large-scale epidemiological studies, the reported prevalence ranges from 5% to 35.7% [3-7]. In clinical practice, images of CMB can guide the diagnosis of diseases such as dementia, stroke, traumatic brain injury, and cerebral amyloid angiopathy [8-11]. Therefore, early and accurate detection of CMBs is essential for developing effective diagnostic and therapeutic strategies.

Traditionally, MRI is primarily used to manually calculate the number and location of CMBs based on their shape, size, and signal characteristics. Clinically, commonly used techniques encompass SWI and gradient echo (GRE). Different centers have different detection techniques. In 2009, Greenberg et al [1] published a consensus on the detection of CMBs. However, at present, manual detection is mainly used. Although manual detection is highly accurate, it relies heavily on expert interpretation and substantial prior knowledge, resulting in increased detection costs. Therefore, there is an urgent need to explore effective auxiliary detection approaches. Deep learning (DL), a class of deep neural networks, has recently attracted attention due to its high performance in image recognition and processing [12]. For traditional machine learning, image segmentation, feature extraction, and feature selection need to be completed before model establishment, which may lead to the loss of critical image information [13]. In contrast, DL can intelligently select features from presegmented images or integrate image segmentation, feature extraction, and selection into the training process to enhance the detection of positive cases [14]. In recent years, DL has been increasingly used in intracerebral hemorrhage (ICH). Several reviews have highlighted its promising performance in detecting ICH [15]. Furthermore, the potential of DL for detecting CMBs has been investigated.

However, there is still a lack of systematic evidence on the accuracy of DL-based models for diagnosing CMBs. Hence, it is challenging to build efficient, intelligent, and assistive diagnostic tools. Accordingly, this study aimed to assess the efficiency of DL-based models for detecting CMBs, thereby providing a theoretical basis for developing and updating AI-based diagnostic techniques.

## Methods

### Study Registration

This study was reported in accordance with the PRISMA (Preferred Reporting Items for Systematic Reviews and Meta-Analyses; Multimedia Appendix 1) guidelines and was prospectively registered in PROSPERO (registration ID CRD42024628447).

### Eligibility Criteria

Textbox 1 presents inclusion and exclusion criteria.

**Eligibility criteria.**

**Inclusion criteria**

- Studies that established deep learning (DL) models for diagnosing cerebral microbleeds (CMBs)
- Studies that reported any of the following outcome measures: confusion matrix, receiver operating characteristic curve (ROC), area under the ROC curve, specificity, sensitivity, precision, accuracy, negative likelihood ratio, positive likelihood ratio, or *F*_1_ score
- Studies published in English

**Exclusion criteria**

- Unpublished conference abstracts
- Studies that only performed image segmentation without constructing DL models for CMBs
- Studies that focused exclusively on the development of machine learning models without incorporating DL models
- Studies that did not assess the diagnostic accuracy of the model

### Data Sources and Search Strategy

IEEE, Web of Science, Embase, the Cochrane Library, and PubMed were comprehensively searched up to November 1, 2024, and the database search was subsequently updated on July 5, 2026, to capture any recently published studies. The search strategy was designed by combining free-text terms and subject headings. Key concepts included two domains: (1) DL and (2) the target disease (eg, “CMB”). There were no restrictions on language, publication date, or study type. Table S1 in Multimedia Appendix 2 presents the complete search strategy.

### Study Selection and Data Extraction

The searched studies were imported into EndNote (Clarivate Analytics). After deduplication, the titles and abstracts of the remaining articles were screened to exclude irrelevant articles. Subsequently, full texts of potentially relevant studies were reviewed to determine eligible articles. Before data extraction, a standardized electronic form was designed. Collected data included country of origin, article title, DOI, year of publication, study type, patient source, first author, task type, imaging modality, diagnostic criteria for CMBs, number of CMBs cases or images, total cases or images, number of CMBs cases or images in training set, total number of cases or images in training set, number of cases or images in testing set, number of CMBs cases or images in testing set, method of validation set generation, number of CMBs cases or images in validation set, number of cases or images in validation set, type of model used, comparison with clinicians (yes/no), and confusion matrix. Two reviewers independently screened articles (κ coefficient=0.891), extracted data, and cross-checked their results. Disagreements were resolved by a third investigator.

### Risk of Bias in Studies

The Quality Assessment of Diagnostic Accuracy Studies-2 (QUADAS-2) tool was used to assess the risk of bias in the eligible studies. It assessed the overall risk of bias and applicability of the original diagnostic tests [16]. The tool included 4 main domains: reference standard, index test, patient selection, and flow and timing. Every domain included several signaling questions, which were answered with “yes,” “unclear,” or “no,” suggesting low, unclear, or high risk of bias, respectively. A study was assessed to have a low risk of bias in a domain when all signaling questions within the domain were answered with “yes.” A “no” response indicated a potential risk of bias. An “unclear” answer suggested that the study did not provide sufficient information for reviewers to make a definitive judgment.

### Synthesis Methods

The meta-analysis of specificity and sensitivity was performed using a bivariate mixed-effects model. Since some original articles did not offer 2×2 diagnostic tables, these tables were reconstructed using sensitivity, specificity, accuracy, and the number of cases by 2 calculation methods (equations 1-4). The bivariate mixed-effects model was used to pool specificity, sensitivity, negative likelihood ratio (NLR), positive likelihood ratio (PLR), diagnostic odds ratio (DOR), and the summary receiver operating characteristic (SROC) curve. Publication bias among studies was tested using Deeks’ funnel plot, while Fagan’s nomogram was used to evaluate the clinical applicability among studies. Subgroup analyses were performed according to the level of analysis (lesion level and patient level) and the method of obtaining the diagnostic 4-fold table at the lesion level (direct extraction and reconstruction).All meta-analyses were performed using Stata software (Stata Corp LLC).




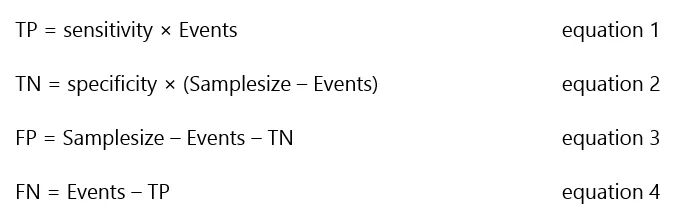




Where, Events represents the number of CMB cases, and Samplesize represents the total number of cases in the corresponding validation set.

## Results

### Study Selection

In total, 462 publications were retrieved from the databases. After the exclusion of 106 duplicates, the titles and abstracts of 356 articles were checked. Among them, 295 articles were deleted for irrelevant topics or study design. The full texts of the remaining 61 publications were browsed. Subsequently, we excluded 1 retracted article, 5 studies without DL, 2 studies that did not differentiate CMBs from other diseases, and 11 unpublished conferences. Ultimately, 42 studies were incorporated in the meta-analysis [17-58] (Figure 1).

**Figure 1:**
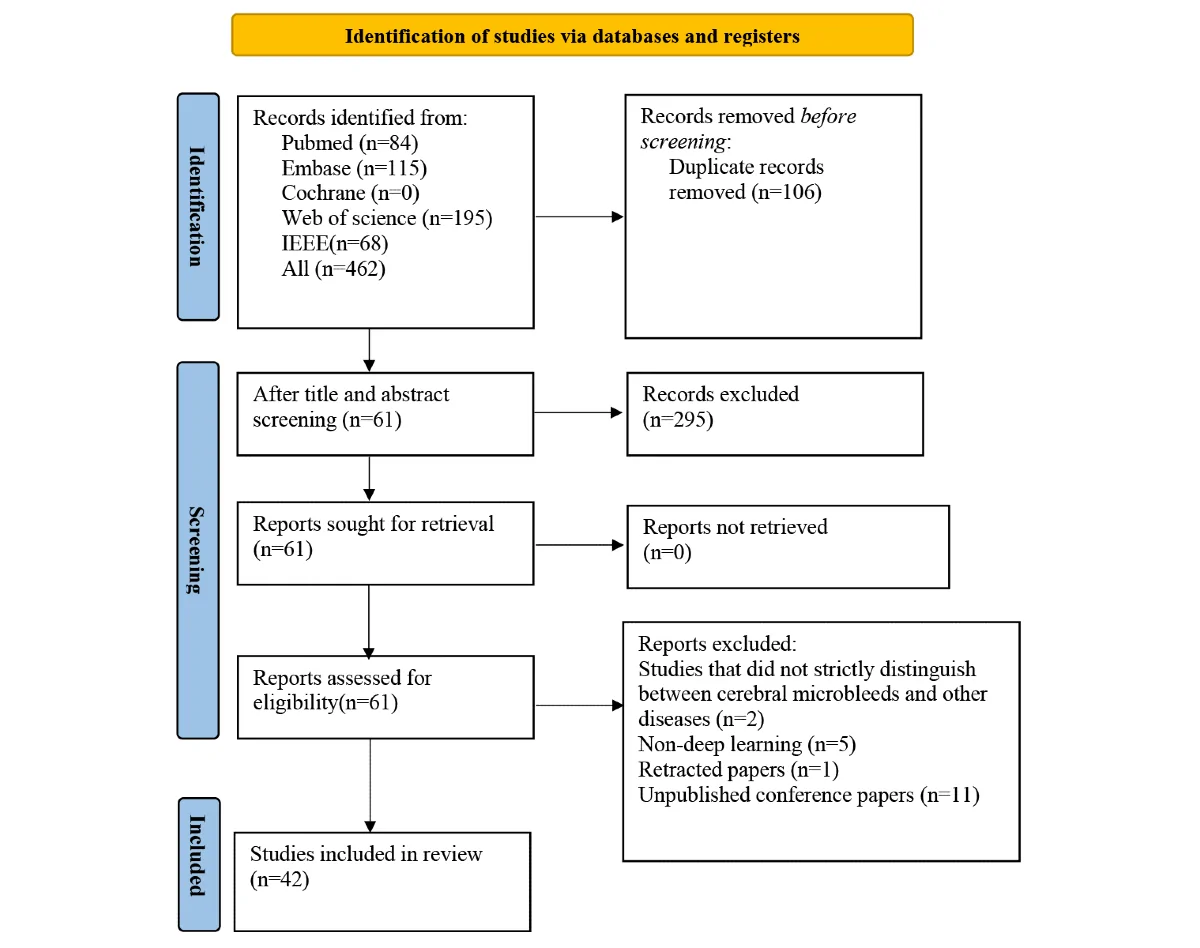
PRISMA (Preferred Reporting Items for Systematic Reviews and Meta-Analyses) flow diagram of literature selection.

### Study Characteristics

The 42 eligible studies were published between 2015 and 2026. All were case-control studies, conducted in 9 countries. Among the 42 studies, 26 were single-center studies; 11 were multicenter studies; 1 involved both a single center and a registry database; 1 was based solely on registry data; 1 involved both multicenter data and a registry database; 1 integrated single-center, multicenter, and registry data; and 1 did not specify data sources. Of the 42 studies, 40 focused on single-classification tasks and 2 were multiclassification tasks. Imaging data were primarily derived from MRI. Specifically, 20 studies used only SWI sequences; 5 studies used both SWI sequences and SWI phase maps; 6 studies used SWI combined with T2*-weighted gradient echo (T2*GRE); 1 study used quantitative susceptibility mapping (QSM), SWI, and T2*GRE sequences; 1 study used QSM alone; 1 study used T2*WI, QSM, and SWI; 1 study used GRE alone; 1 study used susceptibility-weighted sequences; 1 study used susceptibility-weighted angiography sequences; 1 study used SWI, phase maps, and magnetization-prepared rapid gradient-echo (MPRAGE); 1 study used MPRAGE, T1WI, T2*WI, fluid-attenuated inversion recovery, and SWI; 1 study used SWI, T1WI, and T2*GRE; 1 study used SWI and T1WI; and 1 study used T1WI, SWI, and GRE. Regarding the generation of the validation set, 23 studies adopted random sampling; 6 applied k-fold cross-validation; 4 used k-fold cross-validation and external validation; 4 used random sampling and external validation; 1 used external validation; 1 applied leave-one-out and cross-validation methods; 1 applied leave-two-out cross-validation methods; 1 used k-fold cross-validation and random sampling, and 1 used random sampling, external validation, and k-fold cross-validation (Table 1).

**Table 1:** Characteristics of the enrolled studies.

First author and year	Author’s country	Research type	Patient sources	Task type	Image sources	Total number of cases/number of images/total number of lesions	Generation method of the validation set
Peng Xia 2023 [42]	China	Case-control	Single-center	Binary classification	MRI^a^—QSM^b^	N/A^c^	Random sampling
So Yeon Won 2024 [40]	Korea	Case-control	Single-center	Binary classification	MRI—SWI^d^	P^e^=33	External validation
N Nishioka 2024 [34]	Japan	Case-control	Single-center	Binary classification	MRI—SWI, T2*WIs^f^	P=33; I^g^=117	Random sampling
Sitara Afzal 2021 [17]	Korea	Case-control	Single-center	Binary classification	MRI—SWI	P=20	Random sampling
MA Al-Masni 2020 [19]	Korea	Case-control	Single-center	Binary classification	MRI—SWI and phase images	P=72; I=188	K-fold cross-validation
Mohammed A Al-masni 2020 [18]	Korea	Case-control	Single-center	Binary classification	MRI—SWI and phase images	P=179; CMBs^h^=760	K-fold cross-validation
Zeeshan Ali 2023 [20]	Pakistan	Case-control	Single-center	Binary classification	MRI—SWI	N/A	Random sampling
Hao Chen 2015 [43]	China	Case-control	Single-center	Binary classification	MRI—SWI	P=20; I=117	Random sampling
Yicheng Chen 2019 [21]	United States	Case-control	Single-center	Binary classification	MRI—SWI	N/A	Random sampling
Zhengfeng Cheng 2019 [22]	China	Case-control	Multicenter	Binary classification	MRI—SWI	N/A	Random sampling
Qi Dou 2015 [23]	China	Case-control	Single-center	Binary classification	MRI—SWI	N/A	Random sampling
Qi Dou 2016 [24]	China	Case-control	Single-center	Binary classification	MRI—SWI	N/A	Random sampling
Zhongding Fang 2023 [26]	China	Case-control	Single-center	Binary classification	MRI—SWI	P=20	K-fold cross-validation
Haejoon Lee 2022 [28]	Korea	Case-control	Multicenter	Binary classification	MRI—SWI	P=207 (dataset 1=128; dataset 2=79); CMBs=515 (dataset 1=367; dataset 2=148)	Random sampling
Saifeng Liu 2019 [30]	United States	Case-control	Multicenter	Binary classification	MRI—SWI	P=220; CMBs=1641	Random sampling
Min Jae Myung 2021 [33]	Korea	Case-control	Single-center	Binary classification	MRI—GRE^i^	N/A	Random sampling
Pingping Fan 2022 [25]	China	Case-control	Single-center, Multicenter Registry Database	Binary classification	MRI—SWI	P=9387; lesions=10,525	Random sampling and external validation
Berakhah F Stanley 2022 [36]	India	Case-control	Single-center	Binary classification	MRI—SWI	SWI-CMB Dataset: P=320; CMBs=1149; SVS^j^-CMB Dataset: P=179; CMBs=760	K-fold cross-validation
Vaanathi Sundaresan 2023 [37]	United Kingdom	Case-control	Multicenter, registration database, and single-center	Binary classification	MRI—T2-GRE^k^, QSM, and SWI	OXVASC^l^ dataset: P=74; CMBs=366; TICH2 dataset: P=115; CMBs=849	K-fold cross-validation
Aleksandra Suwalska 2022 [38]	Poland	Case-control	Single-center	Binary classification	MRI—SWI	Dataset 1: P=304; CMBs=144; dataset 2 (external validation): P=61	Random sampling and external validation
Shuihua Wang 2019 [39]	China	Case-control	Single-center	Binary classification	MRI—SWI	N/A	Random sampling
Ruizhen Wu 2023 [41]	China	Case-control	Multicenter	Binary classification	MRI—SWS^m^	P=364	K-fold cross-validation
Jun-Ho Kim 2024 [44]	Korea	Case-control	Single-center	Binary classification	MRI—SWI and phase images	P=114; CMBs=365	Random sampling
K Koschmieder 2022 [27]	The Netherlands	Case-control	Single-center	Binary classification	MRI—SWI	P=81	Random sampling
Tianfu Li 2021 [29]	China	Case-control	Single-center	Binary classification	MRI—SWAN images	P=58; I=723; CMBs=1301	Random sampling
Siyuan Lu 2017 [31]	China	Case-control	Single-center	Binary classification	MRI—SWI	P=64; CMBs=6285	Random sampling
Yu Luo 2024 [32]	China	Case-control	Single-center	Binary classification	MRI—SWI	P=265; CMBs=1738	Random sampling
Tanweer Rashid 2021 [35]	United States	Case-control	Registration database	Binary classification	MRI—T2*WI, QSM, and SWI	P=24	Leave-one-out cross-validation
Cong Chen 2024 [45]	China	Case-control	Single-center	Binary classification	SWI	P=600; I=78,000; CMBs:1112	Random sampling
Ami Tsuchida 2024 [46]	France	Case-control	Multicenter	Binary classification	T2*GRE and SWI	N/A	Random sampling and external validation
Tahereh Hassanzadeh 2026 [47]	Australia	Case-control	Multicenter + registry database	Binary classification	T2*GRE and SWI	CMBs=3998	Random sampling and K-fold cross-validation
M Mohsin Jadoon 2025 [48]	Pakistan	Case-control	Multicenter	Binary classification	T2*GRE and SWI	P=979; CMBs=888	K-fold cross-validation and external validation
Behrang Khaffafi 2025 [49]	Iran	Case-control	Unclear	Binary classification	SWI	N/A	Random sampling
Jun-Ho Kim 2025 [50]	Korea	Case-control	Multicenter	Multiclassification	SWI, phase images, MPRAGE^n^	GMC^o^ database: P=128; CMBs=365; SNUH^p^ dataset: P=94; CMBs=311	Random sampling and external validation
Ji Su Ko 2025 [51]	Korea	Case-control	Single-center	Binary classification	SWI, phase image	P=422	Random sampling, k-fold cross-validation and external validation
Huiyu Zhao 2025 [52]	China	Case-control	Single-center	Multiclassification	MPRAGE, T1WI^q^, T2*WI, FLAIR^r^, SWI	P=163	K-fold cross-validation and external validation
Kwon Hwi Cho 2026 [53]	Korea	Case-control	Multicenter	Binary classification	T2*-GRE and SWI	P=506	K-fold cross-validation and external validation
Fengchun Liu 2026 [54]	China	Case-control	Multicenter	Binary classification	SWI, T1-weighted, T2*-weighted	P=549	Random sampling
Zhen Xuen Brandon Low 2026 [55]	Australia	Case-control	Multicenter	Binary classification	SWI and T2*-GRE	P=284	K-fold cross-validation and external validation
Lukas Rau 2026 [56]	Germany	Case-control	Single-center	Binary classification	SWI	P=15; I=15; CMBs=40	Leave-two-out cross-validation
Berakhah F Stanley 2026 [57]	India	Case-control	Multicenter	Binary classification	SWI, T1WI		Random sampling
Soo-Oh Yang 2026 [58]	Korea	Case-control	Single-center	Binary classification	T1-weighted, SWI, GRE	P=758; CMBs=8915	Random sampling

^a^MRI: magnetic resonance imaging.

^b^QSM: quantitative susceptibility mapping.

^c^N/A: not available.

^d^SWI: susceptibility-weighted imaging.

^e^P: number of participants.

^f^T2*WI: T2*-weighted imaging.

^g^I: number of images.

^h^CMB: cerebral microbleed.

^i^GRE: gradient echo.

^j^SVS: designation for the dataset acquired using Siemens 3.0 T Verio and Skyra magnetic resonance imaging scanners.

^k^T2-GRE: T2*-weighted gradient echo.

^l^OXVASC: Oxford Vascular Study.

^m^SWS: susceptibility-weighted magnetic resonance sequence.

^n^MPRAGE: magnetization-prepared rapid gradient-echo.

^o^GMC: Gachon University Gil Medical Center.

^p^SNUH: Seoul National University Hospital.

^q^T1WI: T1-weighted imaging.

^r^FLAIR: fluid-attenuated inversion recovery.

### Risk of Bias in Studies

The 42 eligible studies all used a case-control design and enrolled consecutive or randomly selected cases while avoiding inappropriate exclusions. The case-control design was generally considered to introduce a high risk of bias in routine diagnostic accuracy studies. In studies on image-based DL, this design did not eliminate the impact on model robustness. Because the case and control groups may differ in image acquisition equipment, scanning parameters, or preprocessing procedures, these confounding factors directly impact the model input variables. Furthermore, including only extreme typical cases or healthy controls deviated significantly from clinical target populations. Therefore, the risk of bias was assessed as high in the patient selection domain. As these studies applied supervised DL methods, they were necessarily conducted with knowledge of the reference standard and relied on predefined model criteria. Consequently, the risk of bias in the index test domain was considered low. All included studies used reference standards that can accurately distinguish different disease states. However, regarding blinding during reference standard interpretation, 13 studies used a blinding method; 5 did not use blinding; and 24 did not specify blinding procedures. Hence, the risk of bias in this domain was high or unclear. Given that these were diagnostic accuracy studies, adequate time intervals between the index test and reference standard were maintained. Moreover, each study applied a single reference standard, and all enrolled participants were included in the final analysis. Therefore, no high risk of bias was found in the flow and timing domain. Regarding the consistency between the included patients and the background and review question, 4 studies were considered to have an unclear risk of bias because the medical history of the enrolled patients was not clearly described. Additionally, 33 studies provided only limited outcome data and could not be directly included in the current meta-analysis, resulting in a high risk of bias in this domain. Given that all reference standards used were considered appropriate, no concerns were identified regarding their applicability in clinical practice (Figures 2 and 3).

**Figure 2:**
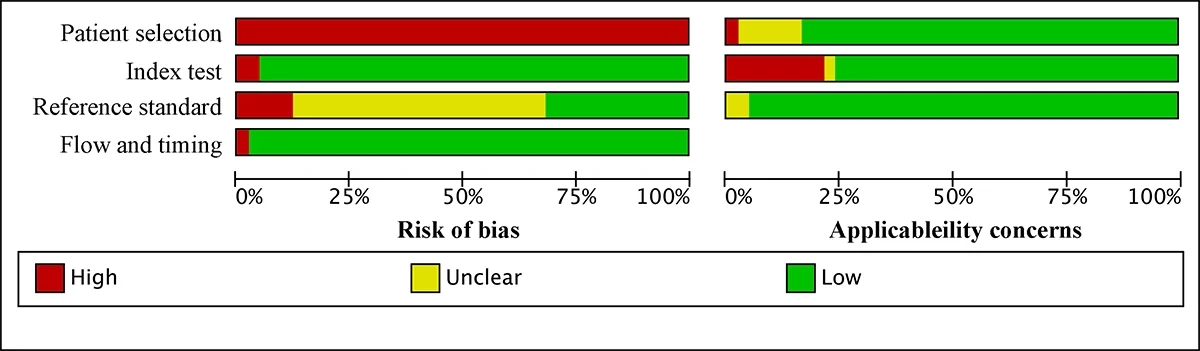
Detailed Quality Assessment of Diagnostic Accuracy Studies-2 assessment results of the enrolled studies.

**Figure 3:**
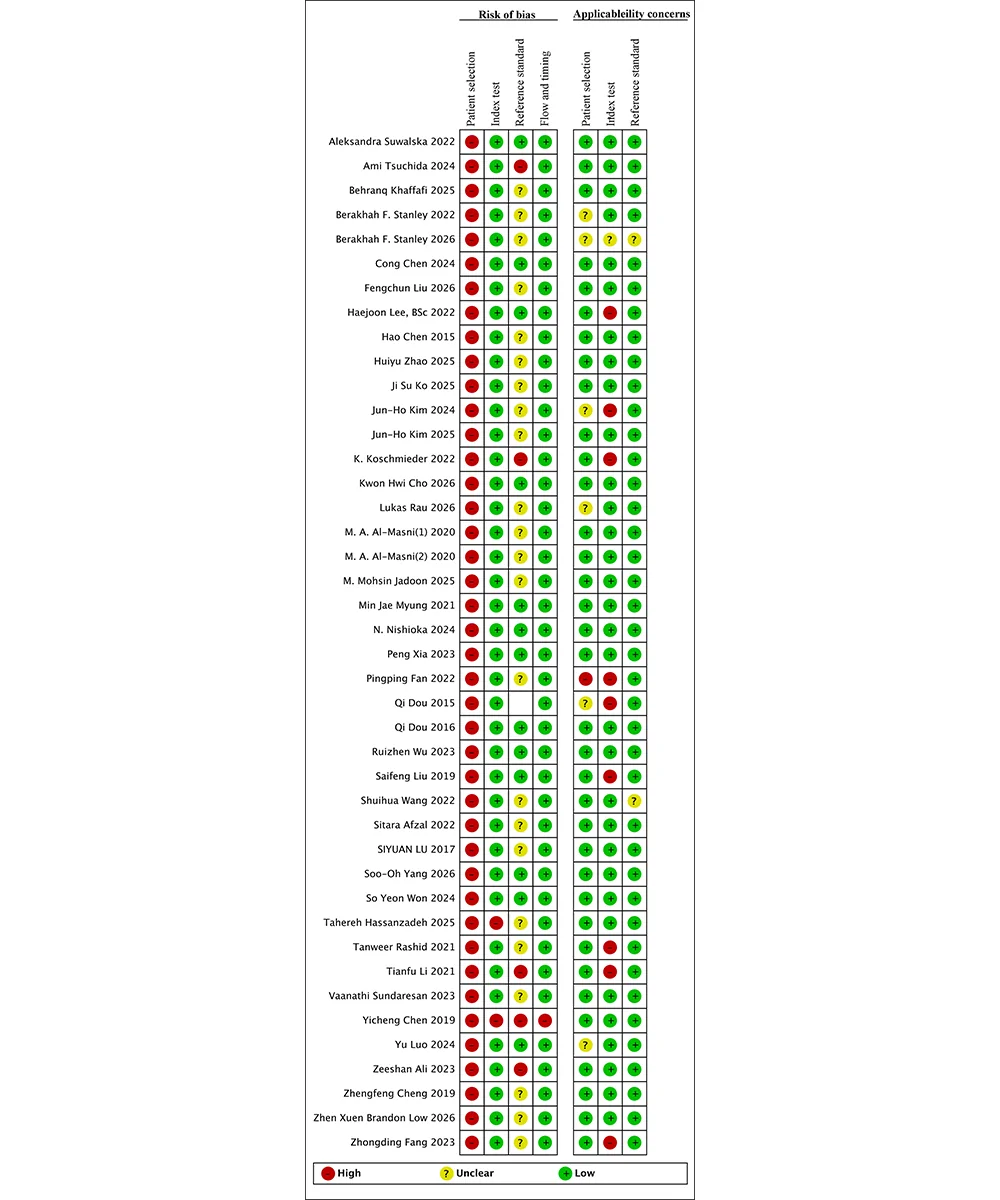
Summary Quality Assessment of Diagnostic Accuracy Studies-2 assessment results of the enrolled studies.

### Meta-Analysis

#### Patient Level

A total of 5 diagnostic 2×2 contingency tables were reported for the performance of DL in detecting CMBs, with the proportion of positive vessels approximately at 32% (134/415). The correlation coefficient was 1.00, suggesting no significant threshold effect. The pooled sensitivity, specificity, PLR, NLR, DOR, and area under the SROC curve (AUC) were 0.89 (95% CI0.76-0.96), 0.86 (95% CI 0.77-0.92), 6.3 (95% CI 3.5-11.6), 0.13 (95% CI 0.05-0.32), 50 (95% CI 12-212), and 0.93 (95% CI 0.91-0.95, respectively (Figures 4 and 5).

**Figure 4:**
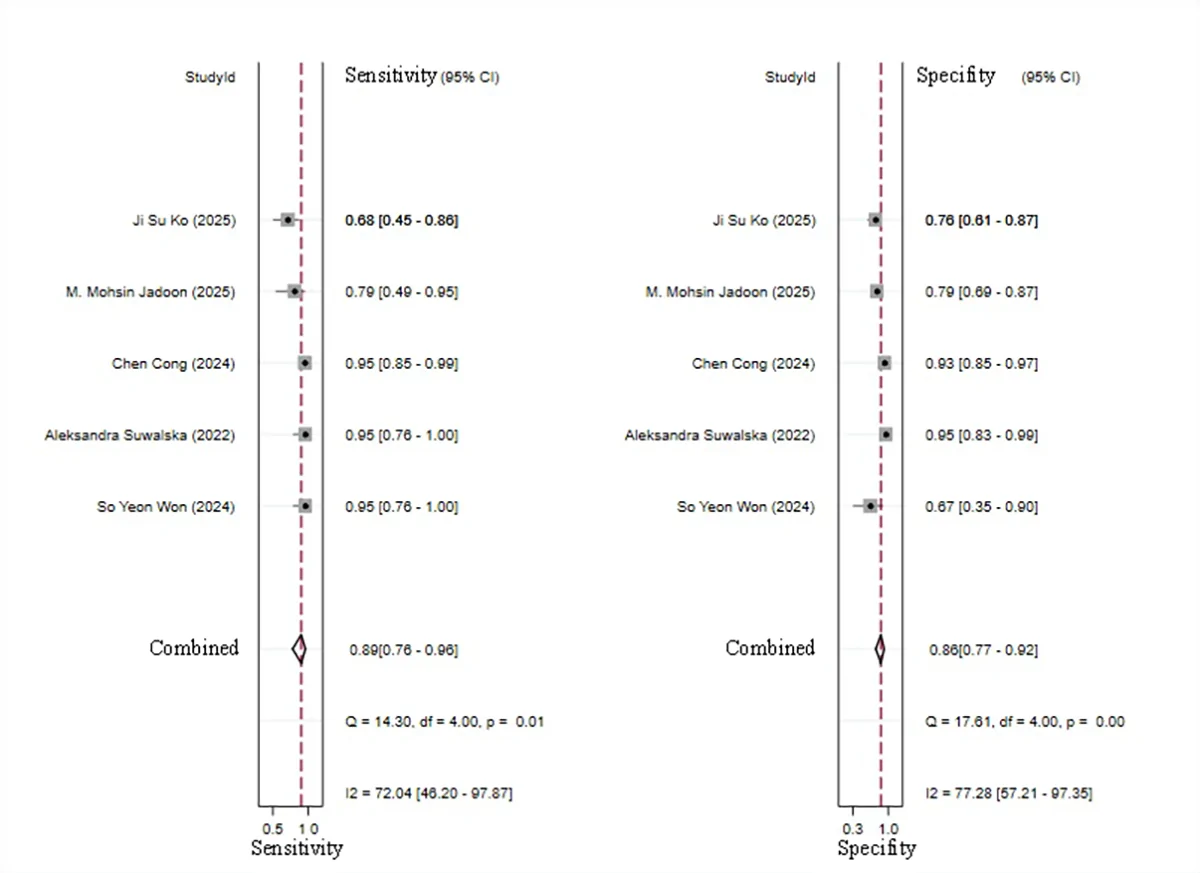
Forest plot of sensitivity and specificity in the meta-analysis of deep learning for cerebral microbleeds detection.

**Figure 5:**
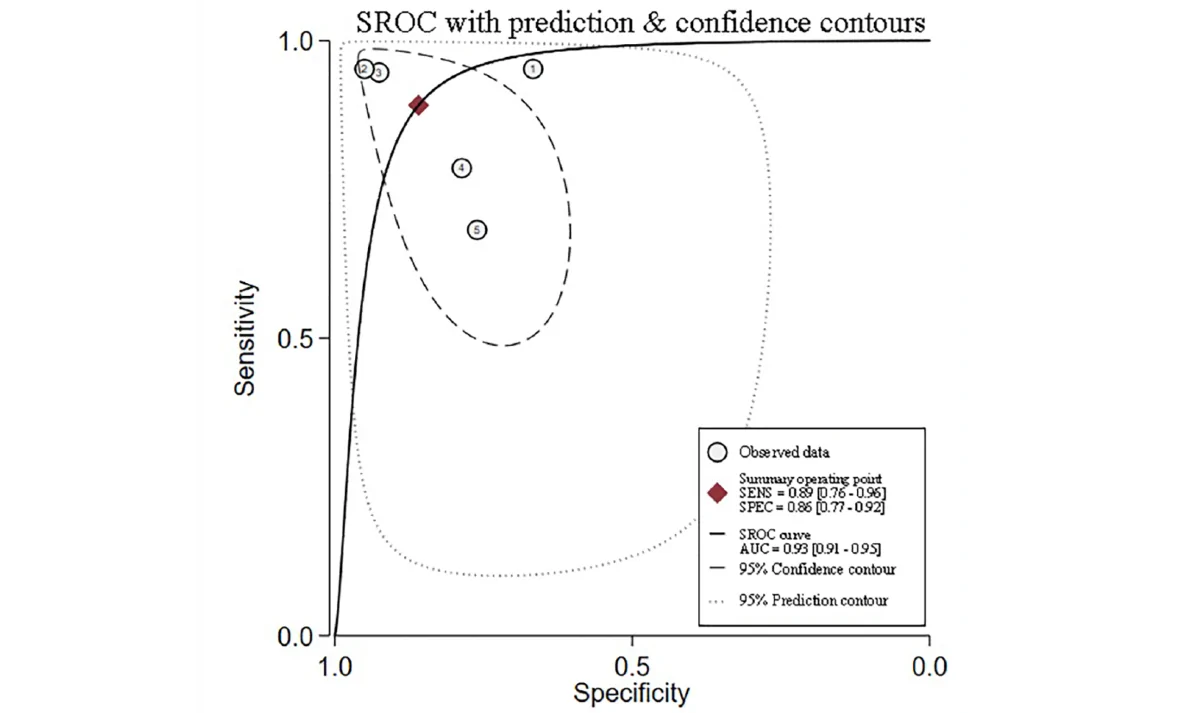
Summary receiver operating characteristic curve of the meta-analysis of deep learning for cerebral microbleeds detection.

Deeks’ funnel plot indicated no significant publication bias (*P*=.37; Figure S1 in Multimedia Appendix 2). Assuming a prior probability of 30%, when the result from a single model was positive, the true positive predictive value (PPV) was 73%. When the result from a single model was negative, the true negative predictive value (NPV) was 95% (Figure S2 in Multimedia Appendix 2).

#### Lesion Level

There were 9 diagnostic 2×2 contingency tables for the performance of DL in detecting CMBs, with the proportion of positive vessels approximately at 37% (12,430/33,909). The correlation coefficient was 0.53, suggesting no significant threshold effect. The pooled sensitivity, specificity, PLR, NLR, DOR, and AUC were 0.96 (95% CI 0.93-0.98), 0.98 (95% CI 0.94-0.99), 39.5 (95% CI 16.8-92.7), 0.04 (95% CI 0.02-0.07), 1061 (95% CI 293-3848), 0.99 (95% CI 0.98-1.00), respectively (Figures S3 and S4 in Multimedia Appendix 2).

Deeks’ funnel plot suggested no significant publication bias (*P*=.51; Figure S5 in Multimedia Appendix 2). Assuming a prior probability of 30%, when the result from a single model was positive, the true PPV was 94%. When the result from a single model was negative, the true NPV was 98% (Figure S6 in Multimedia Appendix 2).

#### Direct Extraction of Diagnostic 4-Fold Tables at the Lesion Level

There were 5 diagnostic 2×2 contingency tables for the performance of DL in detecting CMBs, with the proportion of positive vessels approximately at 45% (11,357/25,248). The correlation coefficient was 0.55, suggesting no significant threshold effect. The pooled sensitivity, specificity, PLR, NLR, DOR, and AUC were 0.98 (95% CI 0.95-0.99), 0.98 (95% CI 0.90-0.99), 40.2 (95% CI 9.3-79.9), 0.02 (95% CI 0.01-0.05), 1738 (95% CI 174-17,385), and 0.99 (95% CI 0.98-1.00) respectively (Figures S7 and S8 in Multimedia Appendix 2).

Deeks’ funnel plot demonstrated no significant publication bias (*P*=.60; Figure S9 in Multimedia Appendix 2). Assuming a prior probability of 30%, when the result from a single model was positive, the true PPV was 95%. When the result from a single model was negative, the true NPV was 98% (Figure S10 in Multimedia Appendix 2).

#### Reconstruction of Diagnostic 4-Fold Tables at the Lesion Level

There were 4 diagnostic 2×2 contingency tables for the performance of DL in detecting CMBs, with the proportion of positive vessels approximately at 12% (1073/8661). The correlation coefficient was 1.00, indicating no significant threshold effect. The pooled sensitivity, specificity, PLR, NLR, DOR, and AUC were 0.95 (95% CI 0.89-0.97), 0.98 (95% CI 0.96-0.99), 41.2 (95% CI 21.2-79.9), 0.06 (95% CI 0.03-0.12), 736 (95% CI 224-2418), and 0.99 (95% CI 0.99-1.00), respectively (Figure S11 and S12 in Multimedia Appendix 2).

Deeks’ funnel plot did not demonstrate significant publication bias (*P*=.51; Figure S13 in Multimedia Appendix 2). Assuming a prior probability of 30%, when the result from a single model was positive, the true PPV was 94%. When the result from a single model was negative, the true NPV was 98% (Figure S14 in Multimedia Appendix 2).

## Discussion

### Summary of the Main Findings

This study systematically evaluated the diagnostic efficacy of DL in diagnosing CMBs from both patient and lesion levels, and further conducted subgroup analyses based on data sources of diagnostic 4-fold tables. Overall, the DL model appeared to have demonstrated relatively favorable diagnostic accuracy in diagnosing CMBs. At the lesion level, the pooled sensitivity, specificity, PLR, and NLR were 0.96 (95% CI 0.93-0.98), 0.98 (95% CI 0.94-0.99), 39.5 (95% CI 16.8-92.7), and 0.04 (95% CI 0.02-0.07), respectively. However, at the more clinically challenging patient level, the pooled sensitivity, specificity, PLR, and NLR were 0.89 (95% CI 0.76-0.96), 0.86 (95% CI 0.77-0.92), 6.3 (95% CI 3.5-11.6), and 0.13 (95% CI 0.05-0.32), respectively. The diagnostic efficacy at the lesion level was better than that at the patient level. This finding suggested that classification tasks based on local image patches were relatively simple, while clinicians should integrate whole-brain information and eliminate many confounding factors to make decisions at the patient level.

### Comparison With Other Previous Reviews

A review by Haller et al [2] has demonstrated that for MRI-based models for CMBs using T2*-weighted or SWI sequences at 1.5T or 3.0T scanners, the true positive rates range from 48% to 89%, and false positive rates vary from 11% to 24%. These results suggest that the diagnostic accuracy of MRI-based models for diagnosing CMB remains suboptimal. Moreover, their findings are reported as ranges only, making it difficult to provide a precise quantitative estimate for readers [2].

Although our review indicated that DL models appeared to exhibit favorable diagnostic accuracy in diagnosing CMBs, there were several challenges. First, 2 image segmentation methods were involved in our study: manual segmentation and automatic segmentation based on DL [59]. In recent years, DL-based automatic segmentation has attracted extensive attention from researchers. For instance, Peng and Sun [59] suggest that the Dice coefficients of DL are 0.90, 0.80, and 0.76 in the segmentation of MRI on brain tumors for the whole tumor, tumor core, and enhancing tumor, respectively. However, segmentation accuracy metrics such as Dice scores were not provided in our included studies. Furthermore, even though they were available in certain articles, the SEs were rarely provided. As a result, we did not quantitatively synthesize segmentation accuracy and instead focused directly on diagnostic performance. Moreover, automatic segmentation methods are preferred for image processing, as manual segmentation is time-consuming and heavily dependent on the prior knowledge of researchers. Future studies should further investigate DL techniques for enhancing the accuracy of MRI-based segmentation of brain lesion areas, which is crucial for developing AI tools and enhancing disease diagnosis.

### Discussion of the Main Findings

This study rigorously distinguished between the patient level and the lesion level to evaluate the diagnostic efficacy of DL models in detecting CMBs. The lesion-level analysis aimed to explore the performance of the model in identifying local image features, while the patient-level analysis more closely reflected whether an individual had CMBs in the actual clinical settings. The results showed that the diagnostic accuracy at the lesion level was superior to that at the patient level. The sensitivity and specificity at the lesion level were 0.96 and 0.98, respectively, with an AUC of 0.99; while the sensitivity and specificity at the patient level were 0.89 and 0.86, respectively, with an AUC of 0.93. The task at the lesion level is relatively simple, making it easier for the model to achieve high accuracy. At the lesion level, DL models typically use image patches as the basic analysis unit, and the models only need to judge whether there are abnormal magnetic susceptibility signals in a local area [47]. The DL models can fully use local spatial information for feature extraction and classification, with fewer interfering factors and clearer tasks. Therefore, its diagnostic efficacy at the lesion level is higher than that at the patient level.

The tasks at the patient level are more complex and more closely resemble real-world clinical scenarios. For the patient-level diagnosis, integrating comprehensive information from hundreds to thousands of image patches across the entire brain is necessary, ultimately determining whether this patient has CMBs. This process is challenging. First, the distribution of CMBs is highly heterogeneous. The model must make robust judgments under uneven lesion distributions [1]. Second, various intracranial signals can simulate microbleed manifestations, including vascular calcification, physiological iron deposition in the basal ganglia, and paramagnetic artifacts. These microbleed-like signals are extremely common in older adults and are difficult to distinguish from true microbleeds on an image [2]. Third, individual differences in image quality (such as motion artifacts and metal artifacts) have amplified their impact at the patient level, as a single low-quality image can interfere with the overall judgment. The above factors may together lead to a decrease in sensitivity and specificity (0.89 and 0.86) at the patient level compared to the lesion level.

The differences in the results between these 2 levels have significant clinical implications. The high diagnostic accuracy at the lesion level suggests that DL models have good diagnostic performance in identifying local lesions and may serve as efficient automated annotation tools for clinical interpretation. However, the diagnostic efficacy at the patient level is less accurate. This suggests that clinical decisions cannot rely solely on the model’s imaging output but must be comprehensively assessed in conjunction with clinical manifestations, vascular risk factors, and the anatomical distribution characteristics of microbleeds. This indicates that DL is still an efficient screening or interpretation tool in the detection of CMBs. Its output still needs to be combined with clinical information and ultimately confirmed by imaging experts, rather than serving as an independent diagnostic basis.

Validation datasets are critical for demonstrating the generalizability of intelligent detection tools. This is especially important in image processing, as image acquisition protocols may vary across institutions and geographic regions, even for the same image modality. If only random sampling is used during the development of DL models, it is difficult to ensure their adaptability across different institutions. As a result, the applicability of such models remains limited [60]. In the study by Yu et al [61], the performance of DL tools declines in the external validation set. Consequently, external validation is imperative for assessing the generalizability of DL tools. In our study, model performance was evaluated according to the method used to generate the validation set. Both cross-validation and random sampling were used for internal validation. Our results demonstrated that the predictive performance metrics, including sensitivity and specificity, were comparable between random sampling and cross-validation. However, models demonstrated lower sensitivity in the external validation set than in the random sampling and cross-validation sets. Therefore, it is crucial to enhance the performance and generalizability of DL models. This study further descriptively summarized the model performance under different validation set generation methods by distinguishing between patient-level and lesion-level. The results showed that the sensitivity reported by a few studies implementing external validation was significantly lower than that of internally validated models in the same studies. However, due to the small number of studies using external validation, and the small number of comparable studies at each level after stratification by patient level and lesion level, subgroup analysis based on validation set generation methods could not be performed. This suggests that our current evidence regarding the accuracy of DL in diagnosing CMBs is largely derived from internal validation. Therefore, future research should prioritize multicenter external validation and standardize the reporting of stratified diagnostic results under different validation sets.

CMBs can be categorized into deep brain types (eg, basal ganglia, thalamus, brain stem, and cerebellum), cortical types (eg, cerebral cortex and juxtacortical areas), and mixed types. These different distribution types are closely related to the pathological mechanisms of CMBs. Deep brain CMBs are often associated with hypertension-associated arteriopathy [62], while cortical CMBs are often associated with coronary arteriosclerosis [11]. Recently, the Boston criteria for diagnosing cerebral amyloid angiopathy v2.0 have been updated, incorporating brain MRI biomarkers to improve diagnostic sensitivity [63]. In the updated criteria, cerebral amyloid angiopathy is defined by strict lobar hemorrhage: ≥2 strict lobar hemorrhage lesions, or ≥1 strict lobar hemorrhage lesion with ≥1 white matter lesion feature. This highlights the importance of the location of CMBs in the diagnosis of cerebral amyloid angiopathy. However, only a few studies report the diagnostic performance of MRI-based DL models for the detection of CMB in different brain regions. Exploring the clinical applicability of MRI-based DL models for the detection of CMB is challenging. Therefore, future research should explore the diagnostic performance of MRI-based DL models in the detection of CMB in different brain regions to guide the development of AI tools for CMB. Although MRI-based DL models showed promising accuracy for the detection of CMB in this study, they cannot be used independently. DL may be used to screen ICH and may serve as an auxiliary tool for clinicians to improve diagnostic efficiency. However, its application in clinical practice still needs to be verified by experienced physicians.

The impact of sample size should be considered during the development of DL models. The robustness of DL models trained on small datasets may often be questioned [64]. DL is a complex, deep neural network architecture, which requires large volumes of imaging data to ensure the robustness of models [65]. The 42 studies included in our meta-analysis adopted only limited image datasets, which may affect the robustness of the developed models. Thus, future research should incorporate images from more centers, diverse ethnic groups, and varied imaging protocols to enhance the performance of DL models and facilitate the development of intelligent detection tools.

This tool assesses bias across 4 domains: patient selection, index test, reference criteria, and flow and timing. It is the most commonly used tool in diagnostic accuracy research. Given that all included studies focused on imaging diagnosis based on supervised DL, we also considered methodological guidelines related to AI. Checklist for Artificial Intelligence in Medical Imaging provides a reporting framework for AI imaging research [66]. QUADAS-AI, developed through the international Delphi consensus, is an extension of QUADAS-2, specifically assessing AI-specific sources of bias, including dataset construction and sourcing, data leakage between training and test sets, image preprocessing, external validation, and model robustness [67,68]. In our study, QUADAS-2 adequately reflected the general methodological quality of the included studies in the patient selection, reference criteria, and flow and timing domains. However, the results should be interpreted with caution because QUADAS-2 did not explicitly assess AI-specific risks, such as the risk of data leakage between the training and validation sets, the adequacy of external validation, and generalizability across scanning devices and acquisition protocols. Since the QUADAS-AI tool was not yet officially available at the start of our evaluation, we did not use it to reevaluate the included studies. Nonetheless, the aforementioned AI-specific methodological issues have been explicitly considered in the process of evaluating the certainty of the evidence.

### Strengths and Limitations of the Study

This study systematically reviewed the performance of DL in detecting CMBs, but several limitations should be noted. First, the included articles were all retrospective case-control studies. Such designs are subject to case selection bias, which may lead to overestimation of the pooled sensitivity and specificity. Therefore, conducting more large-sample, prospective original studies on DL for detecting CMBs is necessary. Furthermore, to further assess whether the data reconstruction process might introduce bias, we conducted a sensitivity analysis on the lesion-level data by separately pooling five 2×2 diagnostic 4-fold tables extracted directly from the original studies without reconstruction. The results showed that the pooled sensitivity, specificity, PLR, NLR, DOR, and AUC were 0.98 (95% CI 0.95-0.99), 0.98 (95% CI 0.90-0.99), 40.2 (95% CI 9.3-79.9), 0.02 (95% CI 0.01-0.05), 1738 (95% CI 174-17,385), and 0.99 (95% CI 0.98-1.00), respectively. These results were consistent with the results of the overall lesion-level analysis, including the 4 reconstructed tables. This finding suggests that the reconstructed data did not significantly affect the pooled estimates, and the overall conclusions remain robust. However, due to the limited number of tables that can be directly extracted, interpreting this sensitivity analysis should be done cautiously. Future original studies should directly report complete diagnostic 4-fold tables to reduce the uncertainty caused by data reconstruction.

At present, CMBs are mainly detected by conventional 1.5T or 3.0T MRI (T2*GRE or SWI). 3.0T has a higher detection rate than 1.5T. Furthermore, 7.0T ultrahigh field strength can further improve the detection rate, but it is rarely used in clinical practice due to its high cost. SWI has a higher detection rate than 2-dimensional GRE, but its high-resolution 3D imaging requires a longer scanning time. Phase imaging, as an inherent sequence of MRI, does not require additional time or cost, and is helpful in differentiating microbleeds from calcification when there is no computed tomography reference. QSM has advantages over SWI, such as quantifying magnetic susceptibility, not relying on specific sequences, eliminating halo effects, and accurately quantifying microbleed volume. It is expected to be included in future diagnostic standards as a quantitative tool. Nonetheless, its clinical application is limited due to technical complexity, high postprocessing threshold, long scanning time, reliance on high field strength, insufficient clinical validation, and a lack of standardized protocols [2]. However, due to the limited number of studies, after we distinguished between the patient and lesion levels, we were unable to conduct subgroup analyses by field strength or sequence to explore the impact of these technical parameters on the accuracy of DL models. Future original studies should standardize the reporting of stratified diagnostic efficacy under different field strength and sequence conditions to more accurately assess the impact of these technical parameters on the diagnostic accuracy of DL models. Nevertheless, considerable clinical and methodological heterogeneity is observed among the included studies across different imaging protocols, study populations, model characteristics, sample sizes, and validation methods. These uncontrolled heterogeneity factors may affect the robustness and generalizability of the pooled estimates. Therefore, generalizing the findings of this study to different clinical settings should be done cautiously.

Among the studies included in our analysis, some studies mainly relied on random sampling and internal validation for image segmentation and validation set generation, with no independent external validation. This limitation may restrict the interpretability of the results. First, the model performance of DL is often affected by parameter settings, which can vary across centers. Therefore, multicenter validation is necessary during the validation process. Two main approaches were used to generate validation datasets: internal validation and external validation. Internal validation methods included leave-one-out, k-fold cross-validation, random sampling, and bootstrap. Notably, these internal validation techniques involved considerable randomness and did not introduce differences between the images in the training and validation sets or diversify the imaging parameters between these sets. As a result, the interpretability of models validated internally was limited, especially in image-based DL. Consequently, it is difficult to demonstrate that a model developed under specific center conditions and parameters can perform well in other centers or under different settings [69,70]. External validation mainly involves prospective studies and multicenter data from different locations. After strictly distinguishing the extracted data according to the patient level and the lesion level, we found that the sample size in the external validation set did not meet the minimum statistical power for subgroup analysis. However, based on our extracted data, the diagnostic power of external validation was lower than that of internal validation. Hence, future research should include more multicenter images from different geographical locations to build a more widely applicable DL tool for the intelligent diagnosis of CMBs. Large volumes of imaging data are required to develop models, particularly DL models. At the same time, original studies need to standardize and record a complete diagnostic 4-fold table to provide calculable raw data for updating evidence and making clinical decisions. If the number of available images is insufficient, it is difficult to ensure the generalizability of the developed DL models.

### Conclusions

DL models based on MRI appear to show favorable accuracy in detecting CMBs. This finding suggests that it seems possible to develop an intelligent detection tool based on DL. However, the number of its external validation sets is still limited. Furthermore, there are still some methodological limitations in our analysis, for example, the lack of prospective studies. Therefore, the results should be interpreted cautiously. Future research should include data from multiple centers in different geographical locations to develop a more robust auxiliary detection tool.

## Appendix

Multimedia Appendix 1 PRISMA 2020 checklist.

Multimedia Appendix 2 Search strategy, forest plots, summary receiver operating characteristic curves, Fagan nomograms, and Deeks funnel plots evaluating deep learning performance for cerebral microbleed detection at the patient and lesion levels, including analyses based on directly extracted and reconstructed 2×2 contingency tables.

## Abbreviations

AUC: area under the summary receiver operating characteristic curve
CMB: cerebral microbleed
DL: deep learning
DOR: diagnostic odds ratio
GRE: gradient echo
ICH: intracerebral hemorrhage
MPRAGE: magnetization-prepared rapid gradient-echo
MRI: magnetic resonance imaging
NLR: negative likelihood ratio
NPV: negative predictive value
PLR: positive likelihood ratio
PPV: positive predictive value
PRISMA: Preferred Reporting Items for Systematic Reviews and Meta-Analyses
QSM: quantitative susceptibility mapping
QUADAS-2: Quality Assessment of Diagnostic Accuracy Studies-2
SROC: summary receiver operating characteristic
SWI: susceptibility-weighted imaging
T2*GRE: T2*-weighted gradient echo
T2*WI: T2*-weighted imaging

## Data Availability

The datasets generated and/or analyzed during this study are available from the corresponding author on reasonable request.
