# Supplementary material for: Accuracy of Deep Learning in Detecting Cerebral Microbleeds: Systematic Review and Meta-Analysis
Source: J Med Internet Res. 2026 Sep 21;28:e95041. doi: 10.2196/95041 (PMC13639419; doi:10.2196/95041)
Supplement: Supplementary file 2 [file jmir_v28i1e95041_app2.docx]

**Supplementary Materials**

**Table S1 Literature search strategy**

**1.Pubmed**

| Search number | Query | Filters and Limits | Results |
| --- | --- | --- | --- |
| #1 | (((((((((((((((((((((((((Cerebral micro-bleed[Title/Abstract]) OR (Cerebral micro-bleeding[Title/Abstract])) OR (Cerebral micro-bleeds[Title/Abstract])) OR (Cerebral micro-haemorrhage[Title/Abstract])) OR (Cerebral micro-hemorrhage[Title/Abstract])) OR (Cerebral micro-hemorrhaging[Title/Abstract])) OR (Cerebral microbleed[Title/Abstract])) OR (Cerebral microbleeds[Title/Abstract])) OR (Cerebral microhaemorrhage[Title/Abstract])) OR (Cerebral microhaemorrhaging[Title/Abstract])) OR (Cerebral microhemorrhage[Title/Abstract])) OR (Cerebral microhemorrhaging[Title/Abstract])) OR (Cerebral microbleeding[Title/Abstract])) OR (Brain micro-bleed[Title/Abstract])) OR (Brain micro-bleeding[Title/Abstract])) OR (Brain micro-bleeds[Title/Abstract])) OR (Brain micro-haemorrhage[Title/Abstract])) OR (Brain micro-hemorrhage[Title/Abstract])) OR (Brain micro-hemorrhaging[Title/Abstract])) OR (Brain microbleed[Title/Abstract])) OR (Brain microbleeds[Title/Abstract])) OR (Brain microhaemorrhage[Title/Abstract])) OR (Brain microhaemorrhaging[Title/Abstract])) OR (Brain microhemorrhage[Title/Abstract])) OR (Brain microhemorrhaging[Title/Abstract])) OR (Brain microbleeding[Title/Abstract]) | No restrictions applied regarding date range, language, or publication type. | 2796 |
| #2 | deep learning[MeSH Terms] | No restrictions applied regarding date range, language, or publication type. | 45528 |
| #3 | (((((((((((((((((((((((((Transfer Learning[Title/Abstract]) OR (Ensemble Learning[Title/Abstract])) OR (artificial intelligence[Title/Abstract])) OR (Hierarchical Learning[Title/Abstract])) OR (Machine Learning[Title/Abstract])) OR (Neural Networks[Title/Abstract])) OR (Neural Network[Title/Abstract])) OR (CNN[Title/Abstract])) OR (AlexNet[Title/Abstract])) OR (VGGNet[Title/Abstract])) OR (ResNet[Title/Abstract])) OR (GoogLeNet[Title/Abstract])) OR (VGG-11[Title/Abstract])) OR (VGG-13[Title/Abstract])) OR (VGG-16[Title/Abstract])) OR (VGG-19[Title/Abstract])) OR (VGG11[Title/Abstract])) OR (VGG13[Title/Abstract])) OR (VGG16[Title/Abstract])) OR (VGG19[Title/Abstract])) OR (ResNet50[Title/Abstract])) OR (ResNet101[Title/Abstract])) OR (ResNet34[Title/Abstract])) OR (ResNet18[Title/Abstract])) OR (Computer aided-diagnosis[Title/Abstract])) OR (Computer aided diagnosis[Title/Abstract]) | No restrictions applied regarding date range, language, or publication type. | 424695 |
| #4 | #2 OR #3 | No restrictions applied regarding date range, language, or publication type. | 442324 |
| #5 | #1 AND #4 | No restrictions applied regarding date range, language, or publication type. | 84 |

2.Cochrane

| Search number | Query | Filters and Limits | Results |
| --- | --- | --- | --- |
| #1 | (Cerebral micro-bleed):ti,ab,kw OR (Cerebral micro-bleeding):ti,ab,kw OR (Cerebral micro-bleeds):ti,ab,kw OR (Cerebral micro-haemorrhage):ti,ab,kw OR (Cerebral micro-hemorrhage):ti,ab,kw | No restrictions applied regarding date range, language, or publication type. | 9 |
| #2 | (Cerebral micro-hemorrhaging):ti,ab,kw OR (Cerebral microbleed):ti,ab,kw OR (Cerebral microbleeds):ti,ab,kw OR (Cerebral microhaemorrhage):ti,ab,kw OR (Cerebral microhaemorrhaging):ti,ab,kw | No restrictions applied regarding date range, language, or publication type. | 189 |
| #3 | (Cerebral microhemorrhage):ti,ab,kw OR (Cerebral microhemorrhaging):ti,ab,kw OR (Cerebral microbleeding):ti,ab,kw OR (Brain micro-bleed):ti,ab,kw OR (Brain micro-bleeding):ti,ab,kw | No restrictions applied regarding date range, language, or publication type. | 33 |
| #4 | (Brain micro-bleeds):ti,ab,kw OR (Brain micro-haemorrhage):ti,ab,kw OR (Brain micro-hemorrhage):ti,ab,kw OR (Brain micro-hemorrhaging):ti,ab,kw OR (Brain microbleed):ti,ab,kw | No restrictions applied regarding date range, language, or publication type. | 38 |
| #5 | (Brain microbleeds):ti,ab,kw OR (Brain microhaemorrhage):ti,ab,kw OR (Brain microhaemorrhaging):ti,ab,kw OR (Brain microhemorrhage):ti,ab,kw OR (Brain microhemorrhaging):ti,ab,kw | No restrictions applied regarding date range, language, or publication type. | 198 |
| #6 | (Brain microbleeding):ti,ab,kw | No restrictions applied regarding date range, language, or publication type. | 3 |
| #7 | #1or#2or#3or#4or#5or#6 | No restrictions applied regarding date range, language, or publication type. | 262 |
| #8 | MeSH descriptor: [Deep Learning] explode all trees | No restrictions applied regarding date range, language, or publication type. | 331 |
| #9 | (deep learning):ti,ab,kw OR (Transfer Learning):ti,ab,kw OR (Ensemble Learning):ti,ab,kw OR (artificial intelligence):ti,ab,kw OR (Hierarchical Learning):ti,ab,kw | No restrictions applied regarding date range, language, or publication type. | 5560 |
| #10 | (Machine Learning):ti,ab,kw OR (Neural Networks):ti,ab,kw OR (Neural Network):ti,ab,kw OR (CNN):ti,ab,kw OR (AlexNet):ti,ab,kw | No restrictions applied regarding date range, language, or publication type. | 6309 |
| #11 | (VGGNet):ti,ab,kw OR (ResNet):ti,ab,kw OR (GoogLeNet):ti,ab,kw OR (VGG-11):ti,ab,kw OR (VGG-13):ti,ab,kw | No restrictions applied regarding date range, language, or publication type. | 71 |
| #12 | (VGG-16):ti,ab,kw OR (VGG-19):ti,ab,kw OR (VGG11):ti,ab,kw OR (VGG13):ti,ab,kw OR (VGG16):ti,ab,kw | No restrictions applied regarding date range, language, or publication type. | 23 |
| #13 | (VGG19):ti,ab,kw OR (ResNet50):ti,ab,kw OR (ResNet101):ti,ab,kw OR (ResNet34):ti,ab,kw OR (ResNet18):ti,ab,kw | No restrictions applied regarding date range, language, or publication type. | 38 |
| #14 | (Computer aided-diagnosis):ti,ab,kw AND (Computer aided diagnosis):ti,ab,kw | No restrictions applied regarding date range, language, or publication type. | 106 |
| #15 | #8or#9or#10or#11or#12or#13or#or14 | No restrictions applied regarding date range, language, or publication type. | 10537 |
| #16 | #7and#15 | No restrictions applied regarding date range, language, or publication type. | 0 |

**3.Embase**

| Search number | Query | Filters and Limits | Results |
| --- | --- | --- | --- |
| #1 | 'cerebral micro-bleed':ab,ti OR 'cerebral micro-bleeding':ab,ti OR 'cerebral micro-bleeds':ab,ti OR 'cerebral micro-haemorrhage':ab,ti OR 'cerebral micro-hemorrhage':ab,ti OR 'cerebral micro-hemorrhaging':ab,ti OR 'cerebral microbleed':ab,ti OR 'cerebral microbleeds':ab,ti OR 'cerebral microhaemorrhage':ab,ti OR 'cerebral microhaemorrhaging':ab,ti OR 'cerebral microhemorrhage':ab,ti OR 'cerebral microhemorrhaging':ab,ti OR 'cerebral microbleeding':ab,ti OR 'brain micro-bleed':ab,ti OR 'brain micro-bleeding':ab,ti OR 'brain micro-bleeds':ab,ti OR 'brain micro-haemorrhage':ab,ti OR 'brain micro-hemorrhage':ab,ti OR 'brain micro-hemorrhaging':ab,ti OR 'brain microbleed':ab,ti OR 'brain microbleeds':ab,ti OR 'brain microhaemorrhage':ab,ti OR 'brain microhaemorrhaging':ab,ti OR 'brain microhemorrhage':ab,ti OR 'brain microhemorrhaging':ab,ti OR 'brain microbleeding':ab,ti | No restrictions applied regarding date range, language, or publication type. | 4202 |
| #2 | 'deep learning'/exp | No restrictions applied regarding date range, language, or publication type. | 122365 |
| #3 | 'deep learning':ab,ti OR 'transfer learning':ab,ti OR 'ensemble learning':ab,ti OR 'artificial intelligence':ab,ti OR 'hierarchical learning':ab,ti OR 'machine learning':ab,ti OR 'neural networks':ab,ti OR 'neural network':ab,ti OR cnn:ab,ti OR alexnet:ab,ti OR vggnet:ab,ti OR resnet:ab,ti OR googlenet:ab,ti OR 'vgg 11':ab,ti OR 'vgg 13':ab,ti OR 'vgg 16':ab,ti OR 'vgg 19':ab,ti OR vgg11:ab,ti OR vgg13:ab,ti OR vgg16:ab,ti OR vgg19:ab,ti OR resnet50:ab,ti OR resnet101:ab,ti OR resnet34:ab,ti OR resnet18:ab,ti OR 'computer aided-diagnosis':ab,ti OR 'computer aided diagnosis':ab,ti | No restrictions applied regarding date range, language, or publication type. | 508388 |
| #4 | #2 OR #3 | No restrictions applied regarding date range, language, or publication type. | 520273 |
| #5 | #1 AND #4 | No restrictions applied regarding date range, language, or publication type. | 115 |

**4.Web of science**

| Search number | Query | Filters and Limits | Results |
| --- | --- | --- | --- |
| #1 | Cerebral micro-bleed (Topic) OR Cerebral micro-bleeding (Topic) OR Cerebral micro-bleeds (Topic) OR Cerebral micro-haemorrhage (Topic) OR Cerebral micro-hemorrhage (Topic) OR Cerebral micro-hemorrhaging (Topic) OR Cerebral microbleed (Topic) OR Cerebral microbleeds (Topic) OR Cerebral microhaemorrhage (Topic) OR Cerebral microhaemorrhaging (Topic) OR Cerebral microhemorrhage (Topic) OR Cerebral microhemorrhaging (Topic) OR Cerebral microbleeding (Topic) OR Brain micro-bleed (Topic) OR Brain micro-bleeding (Topic) OR Brain micro-bleeds (Topic) OR Brain micro-haemorrhage (Topic) OR Brain micro-hemorrhage (Topic) OR Brain micro-hemorrhaging (Topic) OR Brain microbleed (Topic) OR Brain microbleeds (Topic) OR Brain microhaemorrhage (Topic) OR Brain microhaemorrhaging (Topic) OR Brain microhemorrhage (Topic) OR Brain microhemorrhaging (Topic) OR Brain microbleeding (Topic) | No restrictions applied regarding date range, language, or publication type. | 5623 |
| #2 | deep learning (Topic) OR Transfer Learning (Topic) OR Ensemble Learning (Topic) OR artificial intelligence (Topic) OR Hierarchical Learning (Topic) OR Machine Learning (Topic) OR Neural Networks (Topic) OR Neural Network (Topic) OR CNN (Topic) OR AlexNet (Topic) OR VGGNet (Topic) OR ResNet (Topic) OR GoogLeNet (Topic) OR VGG-11 (Topic) OR VGG-13 (Topic) OR VGG-16 (Topic) OR VGG-19 (Topic) OR VGG11 (Topic) OR VGG13 (Topic) OR VGG16 (Topic) OR VGG19 (Topic) OR ResNet50 (Topic) OR ResNet101 (Topic) OR ResNet34 (Topic) OR ResNet18 (Topic) OR Computer aided-diagnosis (Topic) OR Computer aided diagnosis (Topic) | No restrictions applied regarding date range, language, or publication type. | 217144 |
| #3 | #1 AND #2 | No restrictions applied regarding date range, language, or publication type. | 195 |

**5.IEEE**

| Search number | Query | Filters and Limits | Results |
| --- | --- | --- | --- |
| #1 | ("All Metadata":Cerebral microbleed) OR ("All Metadata":Cerebral micro-bleed ) OR ("All Metadata":Cerebral micro-bleeding ) OR ("All Metadata":Cerebral micro-bleeds ) OR ("All Metadata":Cerebral micro-haemorrhage ) OR ("All Metadata":Cerebral micro-hemorrhage ) OR ("All Metadata":Cerebral micro-hemorrhaging ) OR ("All Metadata":Cerebral microbleeds ) OR ("All Metadata":Cerebral microhaemorrhage ) OR ("All Metadata":Cerebral microhaemorrhaging ) OR ("All Metadata":Cerebral microhemorrhage ) | No restrictions applied regarding date range, language, or publication type. | 35 |
| #2 | ("All Metadata":Cerebral microhemorrhaging ) OR ("All Metadata":Cerebral microbleeding ) OR ("All Metadata":Brain micro-bleed ) OR ("All Metadata":Brain micro-bleeding ) OR ("All Metadata":Brain micro-bleeds ) OR ("All Metadata":Brain micro-haemorrhage ) OR ("All Metadata":Brain micro-hemorrhage ) OR ("All Metadata":Brain micro-hemorrhaging ) OR ("All Metadata":Brain microbleed ) OR ("All Metadata":Brain microbleeds ) OR ("All Metadata":Brain microhaemorrhage ) | No restrictions applied regarding date range, language, or publication type. | 26 |
| #3 | ("All Metadata":Brain microhaemorrhaging ) OR ("All Metadata":Brain microhemorrhage ) OR ("All Metadata":Brain microhemorrhaging ) OR ("All Metadata":Brain microbleeding ) | No restrictions applied regarding date range, language, or publication type. | 7 |

**Figure S1** Deeks’ funnel plot of the MA of DL for CMBs detection

**Figure S2** Fagan’s nomogram of the MA of DL for CMBs detection

**Figure S3** Forest plot of the MA of sensitivity and specificity for DL in detecting CMBs under internal validation by random sampling

**Figure S4** SROC curve of the MA of DL in detecting CMBs under internal validation by random sampling

**Figure S5** Deeks’ funnel plot of the MA of DL in detecting CMBs under internal validation by random sampling

**Figure S6** Fagan’s nomogram of the MA of DL in detecting CMBs under internal validation by random sampling

**Figure S7** Forest plot of the MA of sensitivity and specificity for DL in detecting CMBs under internal validation by cross-validation

**Figure S8** SROC curve of the MA of DL in detecting CMBs under internal validation by cross-validation

**Figure S9** Deeks’ funnel plot of the MA of DL in detecting CMBs under internal validation by cross-validation

**Figure S10** Fagan’s nomogram of the MA of DL in detecting CMBs under internal validation by cross-validation

**Figure S11** Forest plot of the MA of sensitivity and specificity for DL in detecting CMBs under internal validation by external validation

**Figure S12** SROC curve of the MA of DL in detecting CMBs under internal validation by external validation

**Figure S13** Deeks’ funnel plot of the MA of DL in detecting CMBs under internal validation by external validation

**Figure S14** Fagan’s nomogram of the MA of DL in detecting CMBs under internal validation by external validation
